# Predictors of distress in female breast cancer survivors: a systematic review

**DOI:** 10.1007/s10549-017-4290-9

**Published:** 2017-05-28

**Authors:** Ania Syrowatka, Aude Motulsky, Siyana Kurteva, James A. Hanley, William G. Dixon, Ari N. Meguerditchian, Robyn Tamblyn

**Affiliations:** 1 Clinical and Health Informatics Research Group, McGill University, Montreal, QC Canada; 2 Department of Epidemiology, Biostatistics and Occupational Health, McGill University, Montreal, QC Canada; 3 Centre de recherche du Centre hospitalier de l’Université de Montréal, Montreal, QC Canada; 4 Department of Mathematics and Statistics, McGill University, Montreal, QC Canada; 5 Arthritis Research UK Centre for Epidemiology, Manchester Academic Health Sciences Centre, The University of Manchester, Manchester, UK; 6 Health eResearch Centre, Farr Institute, Manchester Academic Health Sciences Centre, The University of Manchester, Manchester, UK; 7 Department of Surgery, McGill University Health Centre, Montreal, QC Canada; 8 Department of Oncology, McGill University Health Centre, Montreal, QC Canada; 9 Department of Medicine, McGill University, Montreal, QC Canada

**Keywords:** Breast cancer, Survivorship, Predictor, Distress, Systematic review

## Abstract

**Purpose:**

Unmanaged distress has been shown to adversely affect survival and quality of life in breast cancer survivors. Fortunately, distress can be managed and even prevented with appropriate evidence-based interventions. Therefore, the objective of this systematic review was to synthesize the published literature around predictors of distress in female breast cancer survivors to help guide targeted intervention to prevent distress.

**Methods:**

Relevant studies were located by searching MEDLINE, Embase, PsycINFO, and CINAHL databases. Significance and directionality of associations for commonly assessed candidate predictors (*n* ≥ 5) and predictors shown to be significant (*p* ≤ 0.05) by at least two studies were summarized descriptively. Predictors were evaluated based on the proportion of studies that showed a significant and positive association with the presence of distress.

**Results:**

Forty-two studies met the target criteria and were included in the review. Breast cancer and treatment-related predictors were more advanced cancer at diagnosis, treatment with chemotherapy, longer primary treatment duration, more recent transition into survivorship, and breast cancer recurrence. Manageable treatment-related symptoms associated with distress included menopausal/vasomotor symptoms, pain, fatigue, and sleep disturbance. Sociodemographic characteristics that increased the risk of distress were younger age, non-Caucasian ethnicity, being unmarried, and lower socioeconomic status. Comorbidities, history of mental health problems, and perceived functioning limitations were also associated. Modifiable predictors of distress were lower physical activity, lower social support, and cigarette smoking.

**Conclusions:**

This review established a set of evidence-based predictors that can be used to help identify women at higher risk of experiencing distress following completion of primary breast cancer treatment.

**Electronic supplementary material:**

The online version of this article (doi:10.1007/s10549-017-4290-9) contains supplementary material, which is available to authorized users.

## Introduction

Around 1.67 million new cases of breast cancer were diagnosed worldwide in 2012, accounting for an estimated 25% of new cancer cases in women [1]. Earlier detection of breast tumors through screening mammography in combination with better and more targeted therapies has dramatically improved survival [2]. Medical advances have generated a large cohort of women surviving after completion of primary breast cancer treatment.

Current 5 and 10-year survival rates following breast cancer diagnosis are 87 and 82%, respectively [3]. As a result, both clinicians and researchers are now focusing more efforts on improving quality of life and patient-centered outcomes in survivorship. The National Comprehensive Cancer Network (NCCN) has recognized distress as an important sequela of cancer diagnosis and treatment [4]. Formally, cancer-related distress is defined as “a multifactorial unpleasant emotional experience of a psychological (i.e., cognitive, behavioral, emotional), social, and/or spiritual nature that may interfere with the ability to cope effectively with cancer, its physical symptoms, and its treatment. Distress extends along a continuum, ranging from common normal feelings of vulnerability, sadness, and fears to problems that can become disabling, such as depression, anxiety, panic, social isolation, and existential and spiritual crises” [4]. Unmanaged distress has been shown to negatively impact all-cause and cancer-related morbidity and mortality, as well as quality of life [5].

Identification of distress during survivorship still presents a challenge; it may be unclear when normal feelings of vulnerability, sadness, and fears transition to a point requiring intervention or support. To address this issue, cancer care agencies have recommended that cancer patients be routinely screened for distress at appropriate intervals throughout primary treatment and survivorship, and at important clinical time points including remission, recurrence, progression, and treatment-related complications [4]. However, approximately 37% of breast cancer patients who have transitioned into survivorship will attend two or fewer follow-up visits with an oncologist within the first year following completion of primary treatment [6], limiting the number of opportunities for distress screening and potentially delaying necessary treatment.

An alternative approach could be to identify breast cancer patients at increased risk of developing distress following transition into survivorship. This would allow for targeted intervention to prevent distress, as well as enhanced monitoring to identify prodromal symptoms and early warning signs of distress for timely intervention to mitigate the risk of progression to diagnosable mental health problems. For example, intervention with prophylactic cognitive behavioral therapy (CBT) has been shown to reduce incidence of depression and anxiety in higher-risk cancer patients by half [7]. As a first step in this direction, the objective of this systematic review is to summarize the published literature around predictors of distress in breast cancer survivors.

## Methods

### Study selection

#### Search strategy

Four databases (MEDLINE, Embase, PsycINFO, and CINAHL) were searched for relevant studies published between January 1, 2000 and March 10, 2016. Studies published prior to the year 2000 were excluded since they were not considered to be representative of the current state of distress literature, given significant improvements in breast cancer treatments and survival rates, and increased awareness of mental health challenges in survivorship. Four main concepts of breast cancer, survivorship, predictor, and distress were mapped to the most relevant controlled vocabulary using Medical Subject Headings (MeSH), and free-text terms were added where necessary. Full search strategies are provided in Appendix 1 in electronic supplementary material.

#### Inclusion and exclusion criteria

This systematic review identified studies that measured the presence of distress (via clinical interviews, or distress scales) and evaluated potential predictors of presence of distress in female breast cancer patients who had completed primary treatment (i.e., surgery, chemotherapy, and/or radiotherapy). Therefore, only studies that dichotomized the outcome as the presence or absence of distress were included in the review; articles that used a continuous outcome (e.g., total score on a distress scale) were not included. Distress was broadly defined based on specific mental health diagnoses (i.e., depressive disorders, anxiety disorders, obsessive–compulsive and related disorders, and trauma- and stressor-related disorders), as well as non-specific symptoms (e.g., ‘psychological,’ ‘psychosocial,’ ‘stress,’ and ‘distress’). All study designs were considered (e.g., cross-sectional, prospective cohort, etc.). Studies were excluded if the article did not report original research, or was not published in the English language.

#### Screening and data abstraction

Screening of articles was completed in two stages. First, articles were screened for relevance based on information provided in the title and abstract, and subsequently evaluated for inclusion based on the full text. Two reviewers independently screened articles at each stage (title and abstract: AS and AM; full text: AS and SK). All articles considered eligible for inclusion by at least one reviewer based on the title and abstract screen were submitted for full-text review. Disagreements at the full-text screen were resolved by discussion and consensus between the two reviewers. Kappa scores were calculated to assess interrater reliability. Reference lists of eligible articles were searched to identify additional relevant studies for inclusion in the review.

One reviewer completed data abstraction (AS), which focused on citation information, study design, sample size and patient characteristics, type and prevalence of distress, measurement of distress (i.e., case ascertainment), timing of measurement, and predictors of distress (all predictors evaluated, and predictors significant in univariate and/or multivariate analyses). A second reviewer (SK) checked data abstracted from ten percent of the articles to assess quality of data abstraction, and one omission was identified.

### Evaluation of predictors

Substantial heterogeneity in the formats of predictors (e.g., continuous, or not comparable classification approaches) limited the feasibility of meta-analysis to quantitatively synthesize results on the strength of association between predictors and the presence of distress. Consequently, significance and directionality of associations (i.e., positive, negative, or inconsistent/unspecified) for the most commonly assessed candidate predictors (*n* ≥ 5) as well as predictors shown to be significant (*p* ≤ 0.05) by at least two studies were summarized descriptively. Predictors were evaluated based on the proportion of studies that showed a significant and positive association (in univariate and/or multivariate analyses) with the presence of distress, in an effort to identify patterns to inform future research.

## Results

### Study selection

**Fig. 1:**
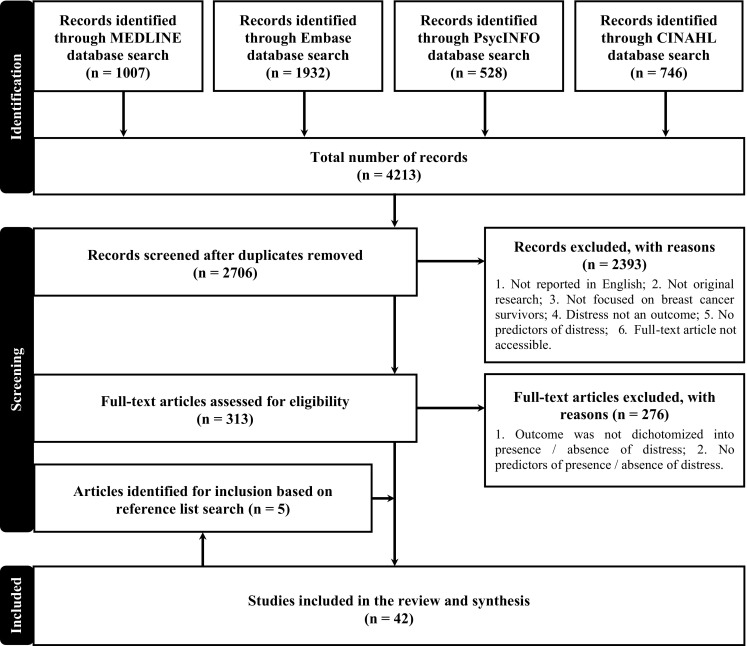
PRISMA study selection flowchart

The search identified 2706 unique articles. The title and abstract screen retained 313 articles. Full-text screening with reference list searching identified 42 studies that met the target criteria and were included in the review. The kappa scores for title and abstract screen, and full-text screen were 0.43 and 0.54, respectively, indicating ‘moderate’ agreement [8]. The moderate kappa scores reflect the complexity around defining distress and uncertainty around the beginning of the breast cancer survivorship period, as well as consideration of studies that did not focus specifically on breast cancer. A modified Preferred Reporting Items for Systematic Reviews and Meta-Analyses (PRISMA) flowchart is presented in Fig. 1 [9].

Characteristics of studies identified through the systematic review are presented in Table 1 [10–51]. Studies were published between 2001 and 2016, and were conducted in North America (19/42 studies; 45%), Asia (12/42 studies; 29%), and Europe (11/42 studies; 26%). Half of the studies collected data using a prospective cohort (21/42 studies; 50%), and the other half used a cross-sectional design (20/42 studies; 48%) or retrospective chart review (1/42 studies; 2%). Eight (8/21 studies; 38%) of the prospective cohort studies reported distress trajectories, which describe how individual women’s distress can change over time from diagnosis through primary treatment and into survivorship. The remaining studies reported prevalence of distress within the survivorship period, without describing how individual women’s distress changes over time.

**Table 1:** Characteristics of studies identified by the systematic review

Author, year (country) [G1–G3: participant groups]	Study design	Sample size	Age, mean ± SD (range) in years^a^	Breast cancer stage	Outcome(s): prevalence (trajectories, if applicable)	Measurement of distress (i.e., case ascertainment)	Timing of distress measurement
Bardwell, 2006 [10] (United States)	Cross-sectional	2595	In survivorship: 53 (28–74)	I–III	Depression: 17%	CES-Dsf ≥ 0.06 (on the logarithmic scale)	≤4 years after completion of primary breast cancer treatment: ≤1 year: 23% 1–2 years: 33% 2–3 years: 24% 3–4 years: 20%
Dominick, 2014 [11] (United States)	Cross-sectional	1817	Not reported	I–III	Depression: No lymphedema—12.2% Lymphedema without lymphedema-related distress—12.8% Lymphedema with lymphedema-related distress—17.6%	CES-Dsf ≥ 0.06 (on the logarithmic scale)	4 years after breast cancer diagnosis
Chen, 2009 [12] (China)	Prospective cohort	1400	At diagnosis: 53.7 ± 9.8	0–IV	Total depression: 26.0% Mild depression: 13.4% Clinical depression: 12.6%	Mild: CES-D = 10–15 Clinical: CES-D ≥ 16	18 months after breast cancer diagnosis
Chen, 2010 [13] (China)	Prospective cohort	1399	At diagnosis: 53.7 ± 9.8	0–III	Total depression: 26.0% Mild depression: 13.4% Clinical depression: 12.6%	Total: CES-D ≥ 10 Mild: CES-D = 10–15 Clinical: CES-D ≥ 16	18 months after breast cancer diagnosis
Kim, 2008 [14] (Korea)	Cross-sectional	1219	In survivorship: 47.4 ± 9.3	0–III	Moderate to severe depression: 24.9%	BDI ≥ 19	Mean ± SD time after breast cancer surgery: 4.6 ± 2.4 years
Mehnert, 2008 [15] (Germany)	Cross-sectional	835	In survivorship: 61.8 ± 9.8 (31–81)	I–IV	Psychological distress (i.e., anxiety, depression, and/or PTSD): 42.9%	HADS ≥ 8 PCL-C = 1 intrusion + 3 avoidance + 2 arousal symptoms (rated ‘moderately’ or above)	Mean ± SD (range) time after breast cancer diagnosis: 46.5 ± 17.5 (18–77) months
Calhoun, 2015 [16] (United States)	Cross-sectional	761	In survivorship: 63.6 ± 10.5	Not reported	Depression: 15.5%	CES-D ≥ 16	Median (range) time after breast cancer diagnosis: 7 (1–43) years
Branstrom, 2015 [17] (Sweden)	Prospective cohort	726	At diagnosis: 51.3 ± 8.1	0–IV	Anxiety: 20.7% Depression: 11.7%	HADS ≥ 8	24 months after breast cancer diagnosis
Saboonchi, 2015 [18] (Sweden)	Prospective cohort; trajectory	725	At diagnosis: 51.2 ± 8.1 (24–63) Median: 52	Not reported	Anxiety trajectories: High stable—6.2% High decrease—15.6% Mid decrease—33.0% Low decrease—45.0%	HADS scores (anxiety subscale): membership in ‘high stable’ trajectory	Over 24 month period following breast cancer surgery
Saboonchi, 2014 [19] (Sweden)	Prospective cohort	654	At diagnosis: 51.3 ± 8.1	Not reported	Anxiety: 25.1% Depression: 15.3%	Total: HADS ≥ 8 Possible: HADS = 8–10 Probable: HADS ≥ 11	12 months after breast cancer surgery
Avis, 2015 [20] (United States)	Prospective cohort; trajectory	653	At diagnosis: 54.9 ± 0.5	I–III	Depression trajectories: 1 consistent very low score—3.8% 2 consistent low score—47.3% 3 consistent borderline score—29.2% 4 high score, declining—11.3% 5 borderline score, increasing—7.2% 6 consistent high score—1.1%	BDI scores: membership in ‘borderline score, increasing’ trajectory	Over 24 month period following breast cancer diagnosis Mean ± SD (range) time since diagnosis at study entry: 4.5 ± 0.05 (6–26) months
Ganz, 2003 [21] (United States)	Cross-sectional	577	At diagnosis: 43.6 (25.2–51) In survivorship: 49.5 (30–61.6)	0–II	Clinical depression: 25.7%	CES-D ≥ 16	Mean ± SD time after breast cancer diagnosis: 5.9 ± 1.5 years Disease-free for 2–10 years
Qiu, 2012 [22] (China)	Cross-sectional	505	In survivorship: 52.02 ± 4.55 (23–65)	0–IV	Major depressive disorder: 20.59%	Phase 1: BDI ≥ 5 Phase 2: MINI Module A (based on DSM-IV criteria)	Mean ± SD (range) time after breast surgery: 17.6 ± 9.0 (6–36) months
Stanton, 2015 [23] (United States)	Prospective cohort; trajectory	457	At diagnosis: 56.4 ± 12.6 (23–91)	I–IV	Depression: 15.6% Depression trajectories: High—38% Recovery—20% Low—32% Very low—11%	CES-D ≥ 16 CES-D scores: membership in ‘high’ trajectory	Over 16 month period following breast cancer diagnosis Mean ± SD time after breast cancer diagnosis at study entry: 2.1 ± 0.8 months
Boehmer, 2012 [24] (United States) [G1: heterosexual women from registry; G2: sexual minority women from registry; G3: sexual minority women from convenience sample]	Cross-sectional	438 G1: 257 G2: 69 G3: 112	In survivorship: G1: 62.7 ± 11.0 G2: 55.9 ± 8.3 G3: 55.1 ± 8.7	0–III	Anxiety: borderline/clinical G1—7.0 / 10.6% G2—6.0 / 8.9% G3—10.8 / 5.4% Depression: borderline/clinical G1—3.1 / 3.1% G2—3.0 / 3.0% G3—6.2 / 4.5%	Borderline: HADS = 8–10 Clinical: HADS ≥ 11	Mean ± SD time after breast cancer diagnosis: G1: 4.7 ± 1.8 years G2: 5.3 ± 1.5 years G3: 6.4 ± 1.8 years
Kim, 2013 [25] (United States)	Cross-sectional	381	Over 21 years old	0–III	Distress (anxiety or depression): not reported	PROMIS: not reported	1–5 years after completion of primary breast cancer treatment
Hong, 2015 [26] (United States)	Prospective cohort	372	Not reported	0–III	Depression: not reported	CES-D > median	1 year after breast cancer diagnosis
Palesh, 2010 [27] (United States)	Prospective cohort	353	In survivorship: 50	Not reported	Time 1: Anxiety: 62% Depression: 15%	Hamilton Anxiety and Depression Scale ≥ 8	Time 1: 6–24 months after primary breast cancer treatment Time 2: 3 months after Time 1
Wang, 2015 [28] (Taiwan)	Prospective cohort; trajectory	311	Not reported	Not reported	Distress trajectories: High depression Medium depression Low depression Depression drop	HADS scores (depression subscale): membership in the ‘high depression’ trajectory	Over 12 month period following breast cancer surgery
Leung, 2016 [29] (Scotland)	Cross-sectional	295	In survivorship: 66.44	Not reported	Psychological distress: 16.6%	GHQ ≥ 4	At least 1 year after breast cancer diagnosis
Romito, 2012 [30] (Italy)	Cross-sectional	255	In survivorship: 58.4 (35–80)	Not reported	Depression: 37%	ZSDS ≥ 60	Mean (range) time since breast cancer diagnosis: 10.5 (5–32) years
Kim, 2013 [31] (Korea) [G1: suicidal ideation present; G2: suicidal ideation not present]	Prospective cohort	241	In survivorship: G1: 49.8 ± 9.6 G2: 50.4 ± 9.8	0–IV	Suicidal ideation: 11.2%	BDI: question about presence of suicidal ideation ≥ 1	1 year after breast cancer surgery
Reyes-Gibby, 2012 [32] (United States)	Cross-sectional	240	In survivorship: 58 ± 16	0–III	Depression: 16.2%	PHQ-8 ≥ 10	Mean (range) time since start of primary breast cancer treatment: 7.9 (6–13) years Median: 8 years
Ashing-Giwa, 2013 [33] (United States)	Cross-sectional	232	In survivorship: 53 ± 10.6 (26–84)	0–III	Clinical depression: 53.4%	CES-D ≥ 16	Time since breast cancer diagnosis: 1–6 years
Wang, 2011 [34] (Taiwan)	Cross-sectional	217	Not reported	Not reported	Distress: not reported	HADS ≥ 15 NCCN Distress Thermometer ≥ 4	Not reported
Lee, 2011 [35] (Korea)	Prospective cohort; trajectory	206	At diagnosis: 47 ± 10	I–III	Depression: 49.3% Deteriorated depressive mood (from breast cancer diagnosis to 1 year following diagnosis): 20.9%	Depression: ZSDS ≥ 50 Deteriorated mood: Effect size > 0.5	Over 1 year period following breast cancer diagnosis
Hsu, 2010 [36] (Taiwan)	Cross-sectional	206	Not reported	I–II	Distress (anxiety or depression): 38.6%	HADS ≥ 15	3–24 months after completion of primary breast cancer treatment
Burgess, 2005 [37] (England)	Prospective cohort	202	At diagnosis: 48.4 ± 7.8	III—32% Other—68%	Depression and/or anxiety annual prevalences: Year 2—25% Year 3—23% Year 4—22% Year 5—15%	SCID for depression and anxiety: standardized diagnostic criteria from the DSM III-R	2–5 years after breast cancer diagnosis
Kornblith, 2001 [38] (United States)	Cross-sectional	179	In survivorship: Median: 56 (32–79)	II	Psychological distress: 8%	MHI ≥ 1.5 SD above the average	Median (range) time since start of chemotherapy: 6.8 (3.3–11.2) years
Henselmans, 2010 [39] (Netherlands)	Prospective cohort; trajectory	171	At diagnosis: 54.8 ± 9.0	0–III	Distress trajectories: No distress—36.3% Recovery—33.3% Late distress—15.2% Chronic distress—15.2%	GHQ scores: membership in ‘late distress’ trajectory	Over 1 year period following breast cancer diagnosis
Accortt, 2015 [40] (United States)	Cross-sectional	163	In survivorship: 47.6 ± 5.6 (28–56)	I–III	Clinical depression: 39%	CES-D ≥ 16	Mean ± SD time following breast cancer diagnosis: 3.4 ± 1.5 years
Donovan, 2014 [41] (United States)	Prospective cohort; trajectory	147	At diagnosis: 51.63 ± 9.03	0–II	Distress trajectories: Class 1 (High)—26.5% Class 2 (Medium)—47.6% Class 3 (Low)—25.9%	CES-D scores: membership in ‘high’ trajectory	Over 12 month period following breast cancer diagnosis
Morasso, 2001 [42] (Italy)	Prospective cohort	132	In survivorship: ≤ 50: 37% 51–60: 35% > 60: 28%	I–III	Psychiatric disorder (major depressive disorder, adjustment disorder, anxiety disorder, dementia, hypomanic episode): 38%	SCID: standardized diagnostic criteria from the DSM III-R	First follow-up visit in first year after start of chemotherapy
Ploos van Amstel, 2013 [43] (Netherlands)	Cross-sectional	129	In survivorship: 57 ± 10	Not reported	Distress: 36%	NCCN Distress Thermometer ≥ 5	Mean ± SD time since breast cancer surgery: 5.6 ± 4.7 years
Kornblith, 2007 [44] (United States) [G1: age ≤55 years; G2: age ≥65 years]	Prospective cohort	128 G1: 61 G2: 67	At diagnosis: G1: 43.6 ± 6.1 G2: 67.1 ± 6.8 In survivorship: G1: 47.9 ± 5.9 (IQR: 43–53) G2: 72.1 ± 5.4 (IQR: 67–76)	I–III	Depression or anxiety: G1—9.8% G2—3.0% PTSD: G1—4.9% G2—0%	HADS ≥ 15 PCL-C = 1 intrusion + 3 avoidance + 2 arousal symptoms (rated ‘moderately’ or above)	Mean ± SD time since completion of primary breast cancer treatment: G1: 3.9 ± 1.65 years G2: 4.5 ± 2.2 years
Brunault, 2013 [45] (France)	Prospective cohort	120	At completion of primary breast cancer treatment: 50.2 ± 8.1 In survivorship: 58.3 ± 8.2	0–IV	Significant depression: 19.2% Possible depression: 12.5% Probable depression: 6.7%	Significant: HADS ≥ 8 Possible: HADS = 8–10 Probable: HADS ≥ 11	Mean ± SD (range) time after completion of primary breast cancer treatment: 8.1 ± 1.3 (6.1–11.0) years
Wang, 2013 [46] (Taiwan)	Prospective cohort; trajectory	Time 1: 248 Time 2: 118	Not reported	Early stages	Distress (anxiety or depression): Time 1—28.63% Time 2—16.10% Distress trajectories: Remained distressed—6% Remained non-distressed—75% Non-distressed to distressed—8% Distressed to non-distressed—11%	HADS ≥ 15	Over a 3 year period: Time 1: ~9 months after completion of primary breast cancer treatment Time 2: ~3 years after Time 1
Eversley, 2005 [47] (United States)	Cross-sectional	116	In survivorship: 47 (29–68)	I–IV	Clinical depression: 52%	CES-D ≥ 16	≤2 years after breast cancer diagnosis and after completion of primary breast cancer treatment
Vahdaninia, 2010 [48] (Iran)	Prospective cohort	99	In survivorship: 46.4 ± 12.5 (24–81)	I–IV	Anxiety: 54.5% Depression: 32.3%	HADS ≥ 8	1 year following completion of primary breast cancer treatment
Neerukonda, 2015 [49] (United States)	Retrospective chart review	81	In survivorship: 53 ± 8	I—43% II—41% Other—16%	Distress: 50%	NCCN Distress Thermometer ≥ 4	First survivorship care visit
Shelby, 2008 [50] (United States)	Prospective cohort	74	In survivorship: Mode: 51 (31–84)	II–III	PTSD: 16.2% Subsyndromal PTSD: 20.3%	SCID PTSD: meet Criterion A, and 1 intrusion + 3 avoidance + 2 arousal symptoms Subsyndromal PTSD: meet Criterion A, and (a) 3 avoidance, or 2 arousal symptoms, or (b) ≥ 5 symptoms across clusters	18 months following breast cancer diagnosis
Baider, 2008 [51] (Israel) [G1: mothers were Holocaust survivors; G2: mothers not Holocaust survivors]	Cross-sectional	39 G1: 20 G2: 19	In survivorship: G1: 46.9 ± 7.1 G2: 46.3 ± 9.8	I–II	Distress: G1—80% G2—32%	GSI ≥ 63	> 6 months after completion of primary breast cancer treatment

*BDI* Beck Depression Inventory, *CES-D* Center for Epidemiologic Studies—Depression scale, *CES-Dsf* CES-D 8-item screening form, *Criterion A* “Actual or threatened death or serious injury, or a threat to the physical integrity of the self or others and a response involving intense fear, helplessness, or horror” [50], *DSM* Diagnostic and Statistical Manual of Mental Disorders, *G1–G3* participant groups (see study-specific descriptions in first column), *GHQ* General Health Questionnaire, *GSI* Global Severity Index, *HADS* Hospital Anxiety and Depression Scale, *IQR* interquartile range, *MHI* Mental Health Inventory, *MINI* Mini International Neuropsychiatric Interview, *NCCN* National Comprehensive Cancer Network, *PCL-C* Posttraumatic Stress Disorder Checklist—Civilian version, *PHQ-8* 8-item Patient Health Questionnaire, *PROMIS* Patient Reported Outcomes Measurement Information System, *PTSD* posttraumatic stress disorder, *SCID* Structured Clinical Interview for DSM, *SD* standard deviation, *ZSDS* Zung Self-rating Depression Scale

^a^ Unless otherwise specified

The majority of studies measured depression (30/42 studies; 71%); anxiety, posttraumatic stress disorder (PTSD), general distress, and suicidal ideation were measured by 29% (12/42 studies), 7% (3/42 studies), 21% (9/42 studies), and 2% (1/42 studies) of studies, respectively. The median prevalence of distress was 26% (interquartile range 39–17 = 22%). The majority of studies assessed the presence of distress using validated cut-offs of the Center for Epidemiologic Studies-Depression scale (CES-D: 12/42 studies; 29%) or the Hospital Anxiety and Depression Scale (HADS: 12/42 studies; 29%). Timing of distress assessment in survivorship varied substantially. Eleven studies (26%) evaluated distress in survivorship at a specific time point following breast cancer diagnosis (ranging from 1 to 4 years). The majority of studies based on distress trajectories (7/8 studies; 88%) followed women for periods ranging from 1 to 2 years starting from breast cancer diagnosis. The remaining studies included survivors with varying times since breast cancer diagnosis, ranging from a mean of 17.6 months following breast surgery (standard deviation (SD): 9.0 months; range 6–36 months) to 10.5 years (range 5–32 years) following breast cancer diagnosis.

### Evaluation of predictors

The significance and directionality of commonly assessed candidate predictors (*n* ≥ 5), as well as predictors shown to be significant (*p* ≤ 0.05) by at least two studies are summarized in Table 2 [10–23, 25, 27–33, 35–50], and categorized based on type of predictor: sociodemographic characteristics, breast cancer characteristics and treatment, treatment-related symptoms, comorbidities and medical history, perceived functioning limitations, and behavioral and support factors. All predictors evaluated within each study, alongside predictors shown to be significant (*p* ≤ 0.05) in univariate and multivariate analyses are presented in Appendix 2 in electronic supplementary material [10–51]. Twenty-eight of the 42 studies (67%) reported on multivariate analyses conducted to estimate independent associations between candidate predictors and the presence of distress in breast cancer survivors; the remaining studies only reported data for univariate associations. Overall, studies that employed a cross-sectional design had larger sample sizes (mean: 560 women vs. 399 women for cohort and chart review studies) and were more likely to report significant associations between candidate predictors and distress.

**Table 2:** Significance and directionality of commonly assessed candidate predictors (*n* ≥ 5), and predictors shown to be significant (*p* ≤ 0.05) by at least two studies

The most commonly evaluated predictors were patient sociodemographic characteristics, breast cancer characteristics, and treatments. Sociodemographic characteristics that were associated with distress included: younger age (10/27 studies; 37%), non-Caucasian ethnicity (2/11 studies; 18%), and being unmarried (8/23 studies; 35%). Lower socioeconomic status (SES) also increased the risk of distress including: lower education (3/21 studies; 14%), lower income (4/7 studies; 57%), and experiencing financial difficulties (5/6 studies; 83%). However, unemployment did not influence the risk of distress.

Breast cancer characteristics and treatments predictive of distress were more advanced cancer at diagnosis (3/21 studies; 14%), treatment with chemotherapy (4/18 studies; 22%), and longer primary treatment duration (2/2 studies). However, type of breast surgery, treatment with radiotherapy, and treatment with hormone therapy did not influence the risk of distress. More recent transition into survivorship (3/10 studies; 30%), and breast cancer recurrence (2/4 studies; 50%) were associated with distress.

The following treatment-related symptoms were associated with distress: menopausal/vasomotor symptoms (7/10 studies; 70%), pain (9/12 studies; 75%), fatigue (6/9 studies; 67%), sleep disturbance (7/9 studies; 78%), lymphedema/arm symptoms (2/5 studies; 40%), breast symptoms (2/3 studies; 67%), appetite loss (2/5 studies; 40%), diarrhea (3/5 studies; 60%), and dyspnea (2/4 studies; 50%). Constipation, nausea, and vomiting did not influence the risk of distress. Furthermore, higher number of treatment-related complaints (3/5 studies; 60%) was associated with distress. Similarly, higher number of comorbidities (5/9 studies; 56%) and history of mental health problems (7/7 studies) increased the risk of distress.

Lower overall quality of life (6/8 studies; 75%) and the following subscales/domains were associated with distress: lower quality of physical health (4/4 studies), lower quality of mental health (2/2 studies), physical functioning limitations (6/8 studies; 75%), role functioning limitations (6/8 studies; 75%), emotional functioning limitations (3/5 studies; 60%), cognitive functioning limitations (2/4 studies; 50%), and social functioning limitations (4/6 studies; 67%). Lower optimism (2/3 studies; 67%), lower posttraumatic growth (3/3 studies), and higher number of stressful life events (3/6 studies; 50%) also increased the risk of distress. In terms of behavioral and support factors, lower physical activity (5/8 studies; 63%), lower social support (6/8 studies; 75%), and cigarette smoking (2/6 studies; 33%) were associated with distress, whereas higher alcohol intake and higher body mass index (BMI) did not influence the risk of distress.

## Discussion

This systematic review is the first synthesis of the published literature around predictors of distress in female breast cancer patients who have completed primary treatment. Breast cancer and treatment-related predictors included more advanced cancer at diagnosis, treatment with chemotherapy, longer primary treatment duration, more recent transition into survivorship, and breast cancer recurrence. Treatment-related symptoms also increased the risk of distress including menopausal/vasomotor symptoms, pain, fatigue, and sleep disturbance. A variety of factors not specific to breast cancer survivors predicted distress. Associated sociodemographic characteristics were younger age, non-Caucasian ethnicity, being unmarried, and indicators of lower SES (specifically, lower education or income, and experiencing financial difficulties). Higher number of comorbidities and history of mental health problems also increased the risk of distress. Furthermore, lower quality of life, optimism, and posttraumatic growth, as well as higher number of stressful life events predicted distress. For behavioral and support factors, lower physical activity, lower social support, and cigarette smoking were associated with distress. Informed by this systematic review, risk stratification may be a viable approach to identify women at higher risk of developing distress following completion of primary breast cancer treatment to provide targeted evidence-based interventions.

Breast cancer-specific factors were commonly evaluated as candidate predictors, given that conventional wisdom suggests that recent, traumatic experiences, such as advanced breast cancer diagnosis associated with worse prognosis and increased risk of premature mortality or more aggressive anti-cancer therapy, may increase the risk of distress. The systematic review identified initial diagnosis of more advanced breast cancer, treatment with chemotherapy, and longer primary treatment duration as predictors of distress. It is difficult to disentangle these predictors, given that they are highly correlated; women with more advanced breast cancer will undergo more aggressive anti-cancer treatment including chemotherapy, which in turn will substantially increase treatment duration. However, a potential underlying mechanism for increased distress in survivorship is that women diagnosed with more advanced breast cancer associated with higher risk of recurrence may experience more intense fears of recurrence [52], which if unmanaged could progress to diagnosable mental health problems. One study included in this systematic review reported significant univariate associations for both breast cancer stage and treatment with chemotherapy with distress; however, only more advanced breast cancer was significant in the multivariate model [31]. Furthermore, the systematic review showed that other forms of anti-cancer therapy (i.e., type of surgery, treatment with radiotherapy, or treatment with hormone therapy) did not influence the risk of distress. These findings are supported by two large Danish cohort studies that evaluated predictors of distress following breast cancer diagnosis and identified number of tumor-positive axillary lymph nodes as an independent predictor of new antidepressant use [53, 54]. Although both studies evaluated breast cancer-related treatments as candidate predictors of distress, neither found independent associations for mastectomy, chemotherapy, or radiotherapy. The results of this systematic review suggest that more advanced breast cancer, as well as its correlates could help to identify women at higher risk of experiencing distress in survivorship.

The review identified potentially modifiable breast cancer treatment-related risk factors. Timely identification and effective management of treatment-related symptoms could serve as a possible intervention to prevent distress or mitigate its effects. Symptoms commonly associated with anti-cancer therapy were predominantly assessed using standardized cancer-specific measures of health-related quality of life as well as breast cancer-specific measures [55, 56]. Other treatment-related symptoms not captured by this systematic review may also be associated with distress. Identification of additional relevant symptoms should be guided through clinical expertise and investigated to assess the relationship with distress. These findings suggest that it may not be anti-cancer therapy that directly affects distress, but rather adverse events resulting from treatment that increase the risk of distress. Uncontrolled chronic and latent treatment-related symptoms can negatively affect health-related quality of life in survivorship and may serve as consistent reminders of the breast cancer diagnosis increasing fear of recurrence [52, 57]. Further studies are needed to assess independent contributions of more advanced breast cancer, treatments, and associated side effects on distress in survivorship.

Additional risk factors not directly related to diagnosis or treatment of breast cancer, including sociodemographic characteristics, comorbidities, medical history, and functional limitations, have also been shown to increase the risk of distress in the general population. In fact, many of these risk factors have been incorporated into predictive algorithms to estimate risk of incident distress in general practice [58–61]. Each of the algorithms includes younger age, indicator(s) of lower SES, and indicator(s) of perceived functioning limitations as predictors. In addition, some algorithms include comorbidities, history of mental health problems, and experiences of discrimination (e.g., racial discrimination [60]). Although this may seem intuitive, the results of this systematic review indicate that risk factors for distress in the general population can also be useful in identifying breast cancer patients at higher risk of distress following completion of primary treatment. Effectively, these risk factors make breast cancer survivors inherently more susceptible to development of distress when faced with challenges in survivorship. However, it is unclear whether or not these factors have differential effects in breast cancer survivors. For example, younger survivors may have different expectations of a normal fulfilling life and experience substantially higher distress as a function of receiving a premature life-threatening diagnosis, as well as coping with potential implications when raising young children. Future studies should focus on identifying interactions between risk factors in the general population and diagnosis of breast cancer in predicting distress.

The review also highlighted modifiable behavior and support factors that could serve as interventions to prevent or mitigate the impact of distress. As expected, lower physical activity, lower social support, and cigarette smoking were associated with the presence of distress [62–64]. In fact, lifestyle and support programs that develop and promote positive coping strategies have been shown to reduce distress symptoms in breast cancer survivors [65–68]. However, contrary to results from prior studies in the general population [69, 70], alcohol intake and BMI did not influence the risk of distress. None of the studies that evaluated alcohol intake showed a significant association. There were low prevalences and absolute numbers of women who reported higher alcohol intake in these studies [10, 13, 35, 50]. Given that higher alcohol intake has been shown to increase risk of breast cancer recurrence [71], this may reflect changes in alcohol consumption due to personal choice or medical advice following breast cancer diagnosis. For studies that reported no association between BMI and distress, three studies compared mean BMI between distressed and non-distressed women, and may have been underpowered to detect significant differences due to lower sample sizes [31, 40, 41]. Another study reported a low prevalence of increased BMI from <25 to ≥25 with a very low number of distressed women transitioning to increased BMI [35]. Future research should focus on exploring these associations in more depth.

This systematic review highlighted an important research gap; no studies evaluated predictors of incident distress in breast cancer survivors. Instead, studies assessed candidate predictors of prevalent distress making it unclear whether the ‘predictor’ or distress occurred first and introducing the possibility of reverse causation. In order to advance this field, future research should focus on establishing predictors of incident distress in breast cancer survivors with no concurrent or recent history of distress. Ideally, a large cohort of breast cancer survivors should be prospectively followed for incident distress, and evidence-based as well as clinically informed candidate predictors should be evaluated using time-to-event analysis.

Furthermore, harmonization of vocabulary around distress and survivorship periods would aid future research to develop more explicit recommendations. First, the non-specific nature of distress makes it difficult to describe and measure. Furthermore, levels and predictors of distress are expected to change across the breast cancer survivorship life course; women who have recently transitioned into survivorship have different concerns and priorities compared to longer-term survivors. Future research should focus on predictors of distress for different intervals of the survivorship period, e.g., transitional survivorship (first year following completion of primary treatment), short-term survivorship (2–5 years after completion of primary treatment), and long-term survivorship (>5 years after completion of primary treatment).

This study has several limitations resulting from the quality and scope of articles identified through the systematic review. Publication bias and inter-study heterogeneity limited the feasibility of conducting predictor-specific meta-analyses. The majority of studies only reported measures of association for significant predictors, which would have biased pooled estimates toward significance. Furthermore, studies that evaluated the same candidate predictor often used different measurements and classification approaches, making predictor-specific meta-analyses impossible. However, the synthesis conducted for this systematic review allowed for direct comparison of significant impact of predictors between studies assessing the same predictor.

This systematic review has established a set of evidence-based predictors that can be used to identify women at higher risk of experiencing distress following completion of primary breast cancer treatment. More advanced breast cancer and treatment-related symptoms may serve as the most practical predictors of distress in survivorship. Furthermore, findings suggest that risk factors for distress in the general population can also be used in this vulnerable population; this intuitively makes sense, given that women predisposed to distress are more likely to experience increased levels as a result of a life-altering breast cancer diagnosis. This systematic review provides preliminary evidence to address an important clinical gap. Furthermore, the results can serve to inform development of a risk stratification algorithm to identify women at higher risk of developing distress following completion of primary breast cancer treatment to provide appropriate support to prevent distress or mitigate its effects.

## Electronic supplementary material

Below is the link to the electronic supplementary material.

Appendix 1 (PDF 77 kb)

Appendix 2 (PDF 172 kb)
