# Supplementary material for: Predictors of distress in female breast cancer survivors: a systematic review
Source: Breast Cancer Res Treat. 2017 May 28;165(2):229–45. doi: 10.1007/s10549-017-4290-9 (PMC5543195; doi:10.1007/s10549-017-4290-9)
Supplement: Supplementary file 1 — Appendix 1 (PDF 77 kb) [file 10549_2017_4290_MOESM1_ESM.pdf]

Appendix 1 MEDLINE, Embase, PsycINFO, and CINAHL systematic review search strategies

Concept	MEDLINE[a]<br><1996 to March 10, 2016>	Embase<br><1996 to 2016 Week 10>	PsycINFO<br><1987 to March Week 2 2016>	CINAHL
Breast cancer	1. exp Breast Neoplasms/<br>2. breast neoplasm*.mp.<br>3. breast cancer*.mp.<br>4. breast tumo?r*.mp.<br>5. or/1-4	1. exp breast tumor/<br>2. breast neoplasm*.mp.<br>3. breast cancer*.mp.<br>4. breast tumo?r*.mp.<br>5. or/1-4	1. Breast Neoplasms/<br>2. breast neoplasm*.mp.<br>3. breast cancer*.mp.<br>4. breast tumo?r*.mp.<br>5. or/1-4	1. (MH "Breast Neoplasms+")<br>2. (MH "Carcinoma, Lobular")<br>3. "breast neoplasm*"<br>4. "breast cancer*"<br>5. "breast tumor*"<br>6. "breast tumour*"<br>7. or/1-6
Survivorship	6. Survivors/<br>7. survivor*.mp.<br>8. or/6-7	6. survivor/<br>7. cancer survivor/<br>8. survivor*.mp.<br>9. or/6-8	6. Survivors/<br>7. survivor*.mp.<br>8. or/6-7	8. (MH "Survivors")<br>9. (MH "Cancer Survivors")<br>10. "survivor*"<br>11. or/8-10
Predictor	9. exp Risk/<br>10. risk*.mp.<br>11. predict*.mp.<br>12. associat*.mp.<br>13. correlat*.mp.<br>14. beta coefficient*.mp.<br>15. odds ratio*.mp.<br>16. rate ratio*.mp.<br>17. hazard ratio*.mp.<br>18. or/9-17	10. exp risk/<br>11. risk*.mp.<br>12. prediction/<br>13. predictor variable/<br>14. predict*.mp.<br>15. associat*.mp.<br>16. correlation analysis/<br>17. correlat*.mp.<br>18. beta coefficient*.mp.<br>19. odds ratio*.mp.<br>20. rate ratio*.mp.<br>21. hazard ratio/<br>22. hazard ratio*.mp.<br>23. or/10-22	9. Risk Factors/<br>10. exp Risk Assessment/<br>11. risk*.mp.<br>12. Prediction/<br>13. predict*.mp.<br>14. associat*.mp.<br>15. exp Statistical Correlation/<br>16. correlat*.mp.<br>17. beta coefficient*.mp.<br>18. odds ratio*.mp.<br>19. rate ratio*.mp.<br>20. hazard ratio*.mp.<br>21. or/9-20	12. (MH "Risk Factors")<br>13. (MH "Risk Assessment")<br>14. "risk*"<br>15. "predict*"<br>16. "associat*"<br>17. (MH "Correlation Coefficient+")<br>18. "correlat*"<br>19. "beta coefficient*"<br>20. (MH "Odds Ratio")<br>21. "odds ratio*"<br>22. (MH "Relative Risk")<br>23. "rate ratio*"<br>24. "hazard ratio*"<br>25. or/12-24
Distress	19. exp Mental Disorders/<br>20. mental.mp.<br>21. mood disorder*.mp.<br>22. Depression/<br>23. depress*.mp.<br>24. dysthymi*.mp.<br>25. Anxiety/<br>26. anxi*.mp.<br>27. phobi*.mp.<br>28. panic disorder*.mp.<br>29. obsessive compulsive disorder*.mp.<br>30. OCD.mp.<br>31. dysmorph*.mp.<br>32. post-traumatic stress disorder*.mp.<br>33. PTSD.mp.<br>34. adjustment disorder*.mp.<br>35. Stress, Psychological/<br>36. stress*.mp.<br>37. distress*.mp.<br>38. psychological.mp.<br>39. psychosocial.mp.<br>40. or/19-39	24. exp mental disease/<br>25. exp mental health/<br>26. mental.mp.<br>27. mood disorder*.mp.<br>28. depress*.mp.<br>29. dysthymi*.mp.<br>30. anxiety/<br>31. anxi*.mp.<br>32. phobi*.mp.<br>33. panic disorder*.mp.<br>34. obsessive compulsive disorder*.mp.<br>35. OCD.mp.<br>36. dysmorph*.mp.<br>37. post-traumatic stress disorder*.mp.<br>38. PTSD.mp.<br>39. adjustment disorder*.mp.<br>40. exp stress/<br>41. stress*.mp.<br>42. distress*.mp.<br>43. psychological.mp.<br>44. psychosocial.mp.<br>45. or/24-44	22. exp Mental Disorders/<br>23. mental.mp.<br>24. mood disorder*.mp.<br>25. "Depression (Emotion)"/<br>26. depress*.mp.<br>27. dysthymi*.mp.<br>28. exp Anxiety/<br>29. anxi*.mp.<br>30. phobi*.mp<br>31. panic disorder*.mp.<br>32. obsessive compulsive disorder*.mp.<br>33. OCD.mp.<br>34. Body Dysmorphic Disorder/<br>35. dysmorph*.mp.<br>36. post-traumatic stress disorder*.mp.<br>37. PTSD.mp.<br>38. adjustment disorder*.mp.<br>39. exp Stress/<br>40. stress*.mp.<br>41. Distress/<br>42. distress*.mp.<br>43. psychological.mp.<br>44. psychosocial.mp.<br>45. or/22-44	26. (MH "Mental Disorders+")<br>27. "mental"<br>28. "mood disorder*"<br>29. (MH "Depression")<br>30. "depress*"<br>31. "dysthymi*"<br>32. (MH "Anxiety+")<br>33. "anxi*"<br>34. "phobi*"<br>35. "panic disorder*"<br>36. "obsessive compulsive disorder*"<br>37. "OCD"<br>38. "dysmorph*"<br>39. "post-traumatic stress disorder*"<br>40. "PTSD"<br>41. "adjustment disorder*"<br>42. (MH "Stress+")<br>43. "stress*"<br>44. "distress*"<br>45. "psychological"<br>46. "psychosocial"<br>47. or/26-46
Overlap of all concepts	41. 5 and 8 and 18 and 40<br>42. limit 41 to English language<br>43. limit 42 to yr="2000 - Current"	46. 5 and 9 and 23 and 45<br>47. limit 46 to English language<br>48. limit 47 to yr="2000 - Current"	46. 5 and 8 and 21 and 45<br>47. limit 46 to English language<br>48. limit 47 to yr="2000 - Current"	48. 7 and 11 and 25 and 47<br>49. 48 (Limiters - Published Date: 20000101-20161231; English Language)

[a]Ovid MEDLINE(R) In-Process & Other Non-Indexed Citations and Ovid MEDLINE(R)
