# Supplementary material for: Predictors of distress in female breast cancer survivors: a systematic review
Source: Breast Cancer Res Treat. 2017 May 28;165(2):229–45. doi: 10.1007/s10549-017-4290-9 (PMC5543195; doi:10.1007/s10549-017-4290-9)
Supplement: Supplementary file 2 — Appendix 2 (PDF 172 kb) [file 10549_2017_4290_MOESM2_ESM.pdf]

Appendix 2 Significant predictors of distress in univariate and multivariate models

**Author, year**	**Predictors evaluated**	**Significant predictors of distress in univariate analysis ($p \leq 0.05$)**	**Significant predictors of distress in multivariate logistic analysis ($p \leq 0.05$)[a]**
Bardwell, 2006 [10]	Treatment (surgery + radiotherapy, surgery + chemotherapy, surgery + both, surgery only); Tamoxifen use; breast cancer stage; time since breast cancer diagnosis; age; marital status; ethnicity; education; BMI; physical activity; alcohol intake; cigarette smoking status; number of NCI dietary guidelines met; physical functioning; pain; vasomotor symptoms; genitourinary symptoms; gastrointestinal symptoms; social support; social strain; optimism; Negative Emotional Expressiveness Questionnaire score; ambivalence over negative emotional expressiveness; hostility; stressful life events; sleep disturbance	Not currently using Tamoxifen; younger age; unmarried; lower education; higher BMI; lower physical activity; cigarette smoking; lower number of NCI dietary guidelines met; physical functioning limitations; pain; vasomotor symptoms; genitourinary symptoms; gastrointestinal symptoms; lower social support; higher social strain; lower optimism; higher ambivalence over negative emotional expressiveness; higher hostility; higher number of stressful life events; sleep disturbance	Model without psychosocial variables: younger age; unmarried; physical functioning limitations; vasomotor symptoms; gastrointestinal symptoms[b]<br>Model with psychosocial variables: lower social support; higher social strain; lower optimism; higher ambivalence over negative emotional expressiveness; higher number of stressful life events; sleep disturbance[c]
Dominick, 2014 [11]	Age; ethnicity; education; marital status; tumor grade; tumor size; menopausal status; number of lymph nodes removed; surgery; chemotherapy; radiotherapy; hormone therapy; BMI; cigarette smoking status; physical activity; comorbidities; lymphedema; lymphedema-related distress	Lymphedema-related distress; age; marital status; BMI; number of lymph nodes removed; comorbidities; cigarette smoking status; physical activity	Younger age; lower number of lymph nodes removed; higher number of comorbidities; cigarette smoking; lower physical activity[d]
Chen, 2009 [12]	Age; education; income; marital status; menopausal status; menopausal symptoms; CCI score; ER/PR status; breast cancer stage; surgery; Tamoxifen use; chemotherapy; radiotherapy; immunotherapy; quality of life (total; physical health summary score; mental health summary score; subscale scores [physical functioning; role limitations due to physical health problems; bodily pain; general health perceptions; vitality (i.e. fatigue); social functioning; role limitations due to emotional problems; and mental health index])	Lower education; lower income; unmarried; menopausal symptoms; higher number of comorbidities; no treatment with radiotherapy; lower quality of life (lower total; lower quality of physical health; lower quality of mental health; lower subscale scores [physical functioning limitations; role limitations due to physical health problems; bodily pain; lower general health perceptions; lower vitality (i.e. fatigue); social functioning limitations; role limitations due to emotional problems; lower mental health index])	Multinomial logistic regression model[e]:<br>Mild depression: higher education; lower income; unmarried (i.e. widowed); lower quality of mental health<br>Clinical depression: lower income; unmarried (i.e. widowed, divorced, separated, single); higher number of comorbidities; no treatment with radiotherapy; lower quality of mental health
Chen, 2010[f] [13]	Exercise participation; duration of exercise; exercise-related MET score; type of exercise; exercise change; tea consumption post-diagnosis (yes, no); tea consumption post-diagnosis (never, former, current); tea consumption amount post-diagnosis (no, yes [≤100 grams/month], yes [>100 grams/month]); lifetime tea consumption, years (no, yes [<18 years], yes [≥18 years]); meat intake; cruciferous vegetable intake; soy food intake; alcohol intake; cigarette smoking status; use of Chinese herbal medicine; total supplement use; ginseng use; ganoderma lucidum capsules/sporophyte use; vitamin supplementation; use of deep sea fish oil pills	Exercise participation (categories not specified); tea consumption (categories not specified)	Shorter duration of exercise; lower exercise-related MET score; exercise change: no exercise (compared to increased exercise level and maintained high exercise level)[g]<br>No tea consumption post-diagnosis (vs. tea consumption); never consumed tea post-diagnosis (vs. current/former tea drinker); no tea consumption post-diagnosis (compared to >100 grams/month); no lifetime tea consumption (compared to tea consumption and <18 years tea consumption)[h]
Kim, 2008 [14]	Age; marital status; education; employment status; income; menopausal status; breast cancer stage; time since breast cancer surgery; local treatments (breast conserving surgery, breast conserving surgery +	Lower education; lower income; menopausal; breast conserving surgery only or mastectomy + radiotherapy as local	Lower income; musculoskeletal disease; dyspnea; insomnia; appetite loss; constipation; arm symptoms[i]

	radiotherapy, mastectomy, mastectomy + radiotherapy); adjuvant treatments (not received, chemotherapy, hormone therapy, chemotherapy + hormone therapy); cerebrovascular disease; cardiac disease; diabetes mellitus; liver disease; lung disease; hypertension; infectious disease; gastrointestinal disease; musculoskeletal disease; kidney disease; nausea and vomiting; pain; dyspnea; insomnia; appetite loss; constipation; diarrhea; breast symptoms; arm symptoms; quality of life/global health status; physical functioning; role functioning; emotional functioning; cognitive functioning; social functioning; body image; sexual functioning; sexual enjoyment; future perspectives	treatments; diabetes mellitus; hypertension; gastrointestinal disease; musculoskeletal disease; pain; dyspnea; insomnia; appetite loss; constipation; diarrhea; breast symptoms; arm symptoms; lower quality of life/global health status; lower for most functional domains (not specified)	
Mehnert, 2008 [15]	Age; education; breast cancer stage; social support; detrimental interactions; time since breast cancer diagnosis	No significant difference for time since breast cancer diagnosis	Younger age; lower education; more advanced breast cancer stage; lower social support; higher level of detrimental interactions[i]
Calhoun, 2015 [16]	Age; ethnicity; marital status; BMI; education; employment status; cigarette smoking status; physical activity; alcohol intake; chemotherapy; radiotherapy; AI use; Tamoxifen use; number of chronic conditions; pain; physical functioning	Ethnicity (being black); number of chronic conditions; pain; current AI use (by black women)	Separate logistic regression models:<br>Black women: current AI use; pain[j]<br>White women: pain[k]
Branstrom, 2015 [17]	Physical activity	Anxiety: lower physical activity<br>Depression: lower physical activity	Anxiety: lower physical activity[l]<br>Depression: lower physical activity[l]
Saboonchi, 2015 [18]	Age; education; having children; living alone; living with husband/partner; born outside Sweden; financial difficulties; mastectomy; axillary clearance; T-classification; N-classification; triple negative breast cancer; chemotherapy; radiotherapy; hormone therapy; physical functioning; role functioning; quality of life/global health status	Not having children; not living with husband/partner; born outside Sweden; financial difficulties; chemotherapy; physical functioning limitations; role functioning limitations; lower quality of life/global health status	Not having children; financial difficulties[m]
Saboonchi, 2014 [19]	Age; education; marital status; radiotherapy; hormone therapy; chemotherapy; sickness absence; adverse life events; history of anxiety at baseline; history of depression at baseline	Anxiety: younger age; history of anxiety at baseline; sickness absence; chemotherapy; experienced adverse life events<br>Depression: history of depression at baseline; sickness absence; chemotherapy; experienced adverse life events	Anxiety: history of anxiety at baseline; experienced adverse life events[n]<br>Depression: history of depression at baseline; experienced adverse life events[n]
Avis, 2015* [20]	Age; marital status; financial difficulties; ethnicity; education; children under 18 years old in the home; first degree family history of breast cancer; breast cancer stage; chemotherapy; hormone therapy; radiotherapy; surgery; vasomotor symptoms; pain; fatigue; spirituality – role of faith; spirituality – meaning/peace; social support; illness intrusiveness	Comparing 'borderline score, increasing' (trajectory 5) vs. 'consistent low score' (trajectory 2): younger age; financial difficulties; not Caucasian; children under 18 years old in the home; more advanced breast cancer stage; chemotherapy; no hormone therapy; vasomotor symptoms; pain; fatigue; lower spirituality – meaning/peace; higher illness intrusiveness	Not reported
Ganz, 2003 [21]	Age	Not significant	Not reported
Qiu, 2012 [22]	Age; marital status; employment status; education; income; family history of mental health problems; personal history of mental health problems; time since breast cancer surgery; surgery; breast cancer stage; ER/PR status; radiotherapy; chemotherapy; immunotherapy; breast cancer recurrence	Unmarried (including separated, divorced, widowed); personal history of mental health problems; breast cancer recurrence	Younger age; unmarried (including separated, divorced, widowed); lower income; personal history of mental health problems; shorter time since breast cancer surgery; breast cancer recurrence[c]
Stanton, 2015 [23]	Age; ethnicity; marital status; income; education; employment status; subjective SES; breast cancer stage; treatment duration; surgery; chemotherapy; radiotherapy; Herceptin; hormone therapy; comorbidities; recruitment site	Comparing 'high' vs. 'low' depression trajectories: younger age; being Latina; not being retired; lower SES; longer treatment duration	Comparing 'high' vs. 'low' depression trajectories: not being retired; higher number of comorbidities

Boehmer, 2012 [24]	Sexual orientation	Not significant	Not reported
Kim, 2013 [25]	Ethnicity (Chinese-American vs. non-Hispanic white); level of acculturation in Chinese-American women; physical activity	Not reported	Lower physical activity[o]
Hong, 2015 [26]	Serum levels of 25-hydroxyvitamin D	Not reported	Lower serum levels of 25-hydroxyvitamin D[p]
Palesh, 2010 [27]	Sleep disturbance	Not reported	Anxiety: sleep disturbance[q]<br>Depression: sleep disturbance[r]
Wang, 2015 [28]	Posttraumatic growth trajectories (i.e. stable high, medium stable, low increasing, low decreasing)	Membership in 'high depression' trajectory: least likely to be member of 'stable high' trajectory for posttraumatic growth	Not reported
Leung, 2016 [29]	Optimism; general health	Lower optimism (general health not evaluated)	Lower general health[s]
Romito, 2012 [30]	Age; education; marital status; time since breast cancer diagnosis; number of comorbidities; treatment (surgery, surgery + radiotherapy, surgery + chemotherapy, and surgery + radiotherapy + chemotherapy); hormone therapy; cigarette smoking status; physical activity; sleep disturbance; fatigue; physical health; mental health	Higher number of comorbidities; sleep disturbance; fatigue; lower physical health; lower mental health	Not reported
Kim, 2013 [31]	Age; education; employment status; marital status; religious; living alone; number of stressful life events; history of depression; HADS scores (for anxiety and depression); number of chronic physical disorders; physical disability; pain; fatigue; BMI; ER+ disease; PR+ disease; tumor size; presence of axillary lymph node; recruitment time since breast cancer diagnosis; chemotherapy; surgery; breast cancer recurrence; breast cancer stage; 5-HTTLPR s allele frequency; STin2 VNTR *10* allele frequency; 5-HTR2a 1438A allele frequency; 5-HTR2a 102C allele frequency; BDNF *met* allele frequency	Unmarried; living alone; higher number of stressful life events; history of depression; higher HADS scores (for anxiety and depression); greater physical disability; fatigue; chemotherapy; breast cancer recurrence; more advanced breast cancer stage; higher STin2 VNTR *10* allele frequency; higher BDNF *met* allele frequency	Living alone; higher HADS score (for anxiety); more advanced breast cancer stage; higher BDNF *met* allele frequency[t]
Reyes-Gibby, 2012 [32]	For inclusion in multivariate model: age; education; marital status; years since breast cancer treatment; metastasis; breast cancer recurrence; diagnosis of new primary cancer; hypertension; heart disease; lung disease; rheumatoid arthritis; osteoarthritis; diabetes; stroke<br>Univariate assessments: cognitive functioning; emotional functioning; role functioning; physical functioning; social functioning; constipation; diarrhea; fatigue; nausea and vomiting; pain; dyspnea; insomnia; loss of appetite; financial difficulties; quality of life/global health status	Cognitive functioning limitations; emotional functioning limitations; role functioning limitations; physical functioning limitations; social functioning limitations; diarrhea; fatigue; nausea and vomiting; pain; dyspnea; insomnia; loss of appetite; financial difficulties; lower quality of life/global health status	Younger age; rheumatoid arthritis; lower number of years since breast cancer treatment[i]
Ashing-Giwa, 2013 [33]	Age; education; place of birth; income; employed prior to breast cancer diagnosis; current employment status; occupation; current marital status; change in marital/relationship status (pre to post breast cancer diagnosis); breast cancer stage; lumpectomy; mastectomy; mastectomy with immediate reconstruction; mastectomy with later reconstruction; radiotherapy; chemotherapy; hormone therapy; physical role limitations; physical well-being; number of comorbidities; neighborhood stress; social support; family stress; functional stress; social functioning limitations; ethnic identity; spirituality	Age (u-shaped association); change in marital/relationship status; lower income; physical role limitations; lower physical well-being; higher number of comorbidities; greater neighborhood stress; lower social support; higher family stress; higher functional stress; social functioning limitations	Not reported
Wang, 2011 [34]	Physical problems listed on the NCCN Distress Thermometer Problem List	18 of 21 physical problems correlated with HADS $\geq$ 15; 16 of 21 physical problems correlated with NCCN Distress Thermometer $\geq$ 4	Not reported
Lee, 2011 [35]	Age; comorbidity; living with spouse; education; religious; employment status; income; physical activity; cigarette smoking status; alcohol intake; ECOG performance status; breast cancer stage; surgery; radiotherapy; chemotherapy; hormone therapy; BMI; change to post-menopausal status;	Change to post-menopausal status; deteriorated financial difficulties; deteriorated emotional support; deteriorated informational support	Change to post-menopausal status; deteriorated emotional support; deteriorated financial difficulties; deteriorated role functioning[u]

	physical functioning; role functioning; emotional functioning; cognitive functioning; social functioning; quality of life/global health status; body image; sexual functioning; future perspectives; fatigue; nausea and vomiting; pain; insomnia; appetite loss; constipation; diarrhea; financial difficulties; hair loss; systemic therapy side effects; breast symptoms; arm symptoms; number of close friends or relatives; tangible support; emotional support; informational support; positive social interaction; affectionate support		
Hsu, 2010 [36]	Breast cancer stage; marital status; perceived support; posttraumatic growth	Not reported	Lower perceived support; lower posttraumatic growth; unmarried (i.e. divorced or widowed)
Burgess, 2005 [37]	Lack of intimate confiding relationship (with a cohabiting partner); age; severe life events; severe non-cancer difficulties; previous episode of depression, anxiety, or both after diagnosis; number of axillary lymph nodes affected; tumor histology; tumor size; adjuvant treatment (none, hormone therapy, chemotherapy, both, or not known)	Not reported	Lack of intimate confiding relationship (with a cohabiting partner); younger age; severe non-cancer difficulties; previous episode of depression, anxiety, or both after diagnosis[v]
Kornblith, 2001 [38]	Education; ethnicity; age; employment; time since beginning of chemotherapy; breast cancer relapse; physical functioning; role functioning; cognitive functioning; emotional functioning; social functioning; quality of life/global health status; fatigue; pain; nausea and vomiting; dyspnea; loss of appetite; insomnia; constipation; diarrhea; financial difficulties; marital status; adequate social support; LES fateful negative events; LES impact of personal illness or injury (past year); LES loss of social support; LES impact of all other negative events (past year); LES impact of positive events (past year); SBI religious; SBI social support	Not reported	Model without psychosocial variables: younger age; no breast cancer relapse; pain[w]<br>Model with psychosocial variables: younger age; shorter time since start of chemotherapy; physical functioning limitations; unmarried (i.e. divorced, separated, or widowed); less than adequate social support; LES greater impact of personal illness or injury (past year)[c]
Henselmans, 2010 [39]	Age; education; breast cancer stage; surgery; adjuvant therapy (radiotherapy only, chemotherapy only, and radiotherapy + chemotherapy); hormone therapy; complaints due to surgery; complaints due to radiotherapy, chemotherapy, and hormone therapy; mastery; optimism; neuroticism	Comparing 'late distress' vs. 'no distress' trajectories: higher number of complaints due to radiotherapy, chemotherapy, and hormone therapy	Comparing 'late distress' vs. 'no distress' trajectories: no significant differences
Accortt, 2015 [40]	Age; years since breast cancer diagnosis; BMI; marital status; employment status; ethnicity; young children in the home; breast cancer treatments received (chemotherapy only, radiotherapy only, both, or neither); surgery; current endocrine therapy; menopausal status change (pre to post after breast cancer treatment); vasomotor symptoms; sleep disturbance	Unemployed; menopausal status change (pre to post after breast cancer treatment); vasomotor symptoms; sleep disturbance	Not reported
Donovan, 2014 [41]	Age; ethnicity; education; marital status; income; menopausal status; BMI; history of depression; CCI score; surgery; breast cancer stage; number of chemotherapy cycles; number of radiation cycles; cumulative radiation dose; hormone therapy; focusing on symptoms; accommodating to illness; maintaining activity; information seeking	Membership in Class 1 (high distress) vs. Class 3 (low distress): unmarried; history of major depression; focusing on symptoms	Multinomial logistic regression: unmarried; focusing on symptoms[x]
Morasso, 2001 [42]	Age; menopausal status; education; history of mental health problems; pathological tumor size; pathological nodes; histology; estrogen receptors; progesterone receptors; surgery	Older age; post-menopausal; history of mental health problems	Older age; history of mental health problems[y]
Ploos van Amstel, 2013 [43]	Time since breast cancer surgery; treatments (surgery only, surgery + radiotherapy, surgery + chemotherapy, surgery + radiotherapy + chemotherapy); HADS scores; helplessness; acceptance; disease benefits; cognitive functioning; emotional functioning; social functioning; physical functioning; role functioning; quality of life/global health status; financial difficulties; dyspnea; pain; fatigue; sleep disturbance; appetite loss; nausea and vomiting; constipation; diarrhea; body image; sexual functioning;	Treatments (surgery + radiotherapy + chemotherapy vs. surgery only); higher HADS scores; increased helplessness; lower acceptance; lower disease benefits; cognitive functioning limitations; emotional functioning limitations; social functioning limitations; physical functioning limitations;	Not reported

	sexual enjoyment; future perspectives; systemic therapy side effects; breast symptoms; arm symptoms; upset by hair loss; vaginal dryness; abnormal blood loss	role functioning limitations; lower quality of life/global health status; financial difficulties; pain; fatigue; sleep disturbance; diarrhea; lower body image; worse future perspectives; systemic therapy side effects; breast symptoms; arm symptoms	
Kornblith, 2007* [44]	Age	No significant differences	Not reported
Brunault, 2013 [45]	Age; time since completion of breast cancer treatment; menopausal status; marital status; breast cancer stage; node status; tumor dimension; histological type; surgery; chemoradiotherapy (sequential or concurrent); hormone therapy; at least one toxicity symptom; pain; edema; fibrosis; telangiectasia; arm lymphedema; atrophy or retraction; ulcer; patient-rated breast cosmetic outcomes [overall cosmetic satisfaction; visibility of the scar; change in skin pigmentation; breast largeness; breast deformation; breast size; breast firmness; nipple displacement]; physician-rated breast cosmetic outcomes [overall cosmetic satisfaction; visibility of the scar]	No significant socio-demographic, cancer-related, or late treatment toxicity variables (breast cosmetic outcomes not reported)	Multinomial logistic regression model[z]:<br>Probable depression vs. no depression: lower patient-rated change in skin pigmentation; patient-rated breast largeness (directionality unspecified); greater patient-rated breast deformation; lower physician-rated overall cosmetic satisfaction<br>Probable depression vs. possible depression: no significant differences
Wang, 2013 [46]	Physical problems; social support; coping styles; posttraumatic growth	Presence of physical symptoms; lower social support; use of negative emotion or cognitive avoidance coping styles; not using positive attitude coping style; lower posttraumatic growth	Not reported
Eversley, 2005 [47]	Ethnicity	Ethnicity (being Latina)	Not reported
Vahdaninia, 2010 [48]	Age; education; marital status; breast cancer stage; fatigue; pain; initial treatment (mastectomy, conservative surgery, chemotherapy, best supportive care)	Not reported	Anxiety: pain[c]<br>Depression: Not being single; fatigue; pain[c]
Neerukonda, 2015 [49]	Age; anxiolytic or antidepressant medication use; other demographic, psychosocial, tumor-related, and treatment characteristics (not described in detail including ethnicity and marital status); factors included in the NCCN Distress Thermometer	Not reported	Younger age[p]
Shelby, 2008 [50]	Socio-demographic variables (not listed); breast cancer stage; surgery; number of stressful life events; number of stressful life events meeting Criterion A; frequency of individual stressful life events (not all listed); physical abuse; rape; history of anxiety disorder; history of pre-cancer PTSD; history of mood disorder; history of alcohol/substance abuse; current mood disorder; current anxiety disorder; current alcohol/substance abuse	Mastectomy (vs. breast conserving surgery); higher number of Criterion A events; physical abuse; history of anxiety disorder; history of pre-cancer PTSD; history of mood disorder; history of alcohol/substance abuse; current mood disorder; current anxiety disorder	Not reported
Baider, 2008* [51]	Mothers were Holocaust survivors	Mothers were Holocaust survivors	Not reported

*Additional calculations were conducted to assess statistical significance of candidate predictors based on data provided in tables: odds ratios with associated 95% confidence intervals were calculated for categorical predictors and t-tests were conducted for continuous predictors; 5-HTR2a: serotonin 2a receptor; 5-HTTLPR: serotonin transporter gene-linked promoter region; AI: aromatase inhibitor; BDNF: brain-derived neurotrophic factor; BMI: body mass index; CCI: Charlson comorbidity index; Criterion A: "Actual or threatened death or serious injury, or a threat to the physical integrity of the self or others and a response involving intense fear, helplessness, or horror." [50]; ECOG: Eastern Cooperative Oncology Group; ER: estrogen receptor; HADS: Hospital Anxiety and Depression Scale; LES: Life Experience Survey; MET: metabolic equivalent; NCCN: National Comprehensive Cancer Network; NCI: National Cancer Institute; PR: progesterone receptor; PTSD: posttraumatic stress disorder; SBI: Systems of Belief Inventory; SES: socioeconomic status; STin2 VNTR: serotonin transporter intron 2 variable number tandem repeat; [a]Bardwell (2006) [10] multivariate analysis used significance of $p \leq 0.001$; [b]Adjusted for predictors significant in the multivariate model ($p \leq 0.001$), treatment (surgery + radiation, surgery + chemotherapy, surgery + both, surgery only), Tamoxifen use, breast cancer stage, time since breast cancer diagnosis, ethnicity, education, BMI, physical activity, alcohol intake, smoking status, number of NCI dietary guidelines met, pain, and genitourinary symptoms; [c]Adjusted for all predictors evaluated; [d]Adjusted for predictors significant in univariate analysis ($p \leq 0.05$), and marital status; [e]Adjusted for predictors significant in univariate analysis ($p \leq 0.05$); [f]Chen (2010) uses an almost identical cohort to Chen (2009). Therefore, predictors tested and reported by both Chen (2009) and Chen (2010) are only recorded under Chen (2009) to avoid double counting; [g]Adjusted for age at diagnosis, education, income, marital status, comorbidity, tea consumption, menopausal symptoms, relapse/metastasis, radiotherapy, and quality of life (short-form 36-item mental health index score); [h]Adjusted for age at diagnosis, education, income, marital status, exercise, comorbidity, menopausal symptoms, relapse/metastasis, radiotherapy, and quality of life (short-form 36-item mental health index score); [i]Adjusted for predictors significant in the

multivariate model ($p \leq 0.05$); [j]Adjusted for predictors significant in the multivariate model ($p \leq 0.05$), and age; [k]Adjusted for AI use, and age; [l]Adjusted for age, education, tumor stage, body mass index, marital status, type of surgery, lymph node involvement, radiotherapy, chemotherapy, and hormonal therapy; [m]Adjusted for significant predictors in multivariate model ($p \leq 0.05$), age, not living with husband/partner, born outside Sweden, and treatment with chemotherapy; [n]Adjusted for predictors significant in the multivariate model ($p \leq 0.05$), age, sickness absence, and treatment with chemotherapy; [o]Adjusted for covariates, e.g. body mass index and comorbidity; [p]Not reported; [q]Adjusted for age; [r]Adjusted for gender, and age; [s]Adjusted for optimism, age, time since breast cancer diagnosis, survey year, socioeconomic status, education, marital status, body mass index, smoking status, and alcohol consumption; [t]Adjusted for predictors significant in multivariate model ($p \leq 0.05$), marital status, number of stressful life events, history of depression, HADS score (for depression), physical disability, pain, fatigue, recruitment time since breast cancer diagnosis, treatment with chemotherapy, breast cancer recurrence, and STin2 VNTR *10* allele frequency; [u]Adjusted for predictors significant in the multivariate model ($p \leq 0.05$), comorbidity, age, radiotherapy, and smoking status; [v]Adjusted for predictors significant in multivariate model ($p \leq 0.05$), and severe life events; [w]Adjusted for predictors significant in multivariate model ($p \leq 0.05$), education, ethnicity, employment, time since beginning of chemotherapy, and physical function; [x]Adjusted for predictors significant in multivariate model ($p \leq 0.05$), and history of depression; [y]Adjusted for predictors significant in the multivariate model ($p \leq 0.15$); [z]Adjusted for predictors significant in the multinomial multivariate model ($p \leq 0.05$), patient-rated overall cosmetic satisfaction, patient-rated visibility of the scar, patient-rated breast size, patient-rated breast firmness, patient-rated nipple displacement, physician-rated visibility of the scar, age, tumor stage at diagnosis, time since completion of treatment, and marital status.
